# Three Dimensional Osteometric Analysis of Mandibular Symmetry and Morphological Consistency in Cats

**DOI:** 10.3389/fvets.2018.00157

**Published:** 2018-07-12

**Authors:** Peter Southerden, Richard M. Haydock, Duncan M. Barnes

**Affiliations:** ^1^Department of Dentistry and Oral Surgery, Eastcott Veterinary Referrals, Swindon, United Kingdom; ^2^The WALTHAM Centre for Pet Nutrition, Melton Mowbray, United Kingdom; ^3^Department of Orthopaedic Surgery, Eastcott Veterinary Referrals, Swindon, United Kingdom

**Keywords:** three dimensional, osteometric, symmetry, morphological, cat, mandibular, facial, asymmetry

## Abstract

**Objectives:** The aim of this study was to describe a number of anatomical reference points which can be used to measure mandibular morphology and assess the degree of mandibular symmetry in a group of normal cats. Comparisons were then made between cats to evaluate correlations between morphological measurements and degree of inter-cat variation. This will provide data valuable evaluating and developing techniques for caudal mandibular fracture repair.

**Methods:** Twenty-seven mixed breed cats (26 Domestic Shorthaired and 1 Domestic Longhaired) with no history of head trauma, intact undamaged mandibles, both mandibular fourth premolars and first molars present which had a full skull CT scan were included in this study. Anatomical reference points were defined on maximum intensity projections of multiplanar reconstruction of the mandibles and measurements taken. The ratios between paired right and left measurements, and the ratio of jaw widths at the coronoid process and mandibular foramen were calculated. All analyses were performed using R version 3.3.3 and the *multcomp* library.

**Results:** None of the right:left ratios were detected as being significantly different from 1 and the coefficient of variation values were all very small showing that when cats deviate from the mean ratio they do so by only a small amount. Measurements analyzed to determine how consistent individual measurements were between cats showed that the most consistent measurement was the lateral ramus inclination angle. The least consistent measurements were ramus height and jaw width at the mental foramen. The correlation between pairs of measurements of the right and left ramus was analyzed and demonstrated a strong correlation between the height, width and length of the ramus.

**Conclusions:** This study has demonstrated a low level of asymmetry between contralateral mandibles in cats, a high level of consistency in the dimensions of mandibles between cats and a strong correlation between the height, width and length of the ramus.

## Introduction

Facial asymmetry is a significant and well-studied condition in humans with a variety of congenital and traumatic causes. Reports of congenital facial asymmetry in cats are rare (18, 19), most cases of asymmetry being the result of jaw fracture (20) secondary to head trauma.

Currently caudal mandibular fractures are most commonly repaired using inter-arcade fixation or stabilization techniques (21). These techniques don't rely on anatomical fracture reduction and are associated with a variety of common complications.

Computed tomography (CT) imaging allows accurate representation of skeletal structures in three dimensions eliminating superimposition and allowing accurate and precise identification of anatomical landmarks (22). CT has been shown to be an accurate technique for measuring skull morphology (6).

The aims of this study were to describe a number of referential measures of mandibular morphology, assess the degree of mandibular symmetry in a group of normal cats, evaluate the correlations between morphological measurements and measure the degree of variation in each measurement between cats. The clinical relevance of this study is to provide data valuable for evaluating and developing techniques for caudal mandibular fracture repair in cats. If cats exhibit a low level of mandibular asymmetry then it will be possible to use the mirror image of an intact mandible for planning and evaluating the accuracy of fixation of a fractured contralateral mandible. Demonstration of consistency of the dimensions of mandibles in cats may facilitate the development of standardized pre-contoured locking plates for caudal mandibular fracture repair.

## Materials and methods

The medical records of cats that presented to Eastcott Referrals, Swindon, UK between 2015 and 2017, were examined. Twenty-eight cats with no history of head trauma, intact undamaged mandibles, both mandibular fourth premolars and first molars present which had a full skull CT scan were included in this study.

CT scans were performed obtaining transverse, 0.625 mm collimated images using a Lightspeed 4 (GE Healthcare, Amersham, UK) CT scanner with kVp of 120 and auto-mA. All CT scans were obtained with patients under general anesthetic.

**Figure 1:**
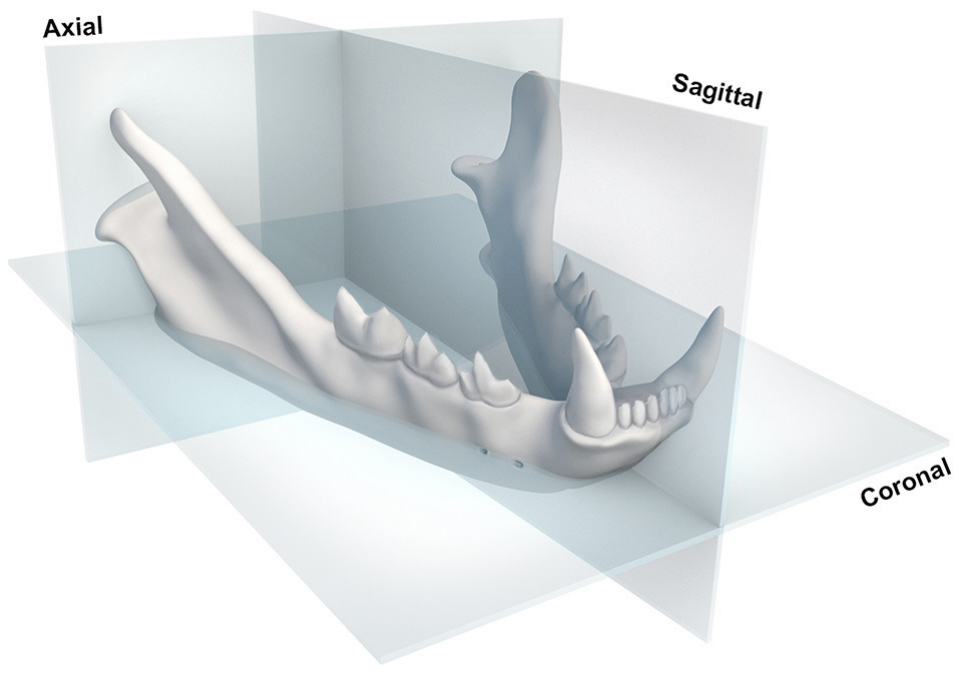
Diagramatic representation of the sagital, axial and coronal reference planes used in this study.

Data for the initial osteometric study was obtained using the DICOM viewing software Horos (The Horos Project, version 2.2.0), which enables multiplanar reformatting and simultaneous viewing of CT images in three orthogonal planes (coronal, sagittal and axial). The CT images were viewed using the bone reconstruction filter. A multiplanar reconstruction (MPR) of the mandibles was performed and orientated to ensure consistent positioning of each scan. The sagittal plane was orientated to intersect the mandibular symphysis between the right and left mandibular first incisors. The axial plane was orientated to intersect the most lateral aspect of the right and left mandibular condyles. The coronal plane was orientated to intersect the middle mental foramen and the most caudal aspect of the angular process (Figure 1). Maximum intensity projections (MIP) of sufficient thickness were created to allow clear visualization of the anatomical landmarks.

**Figure 2:**
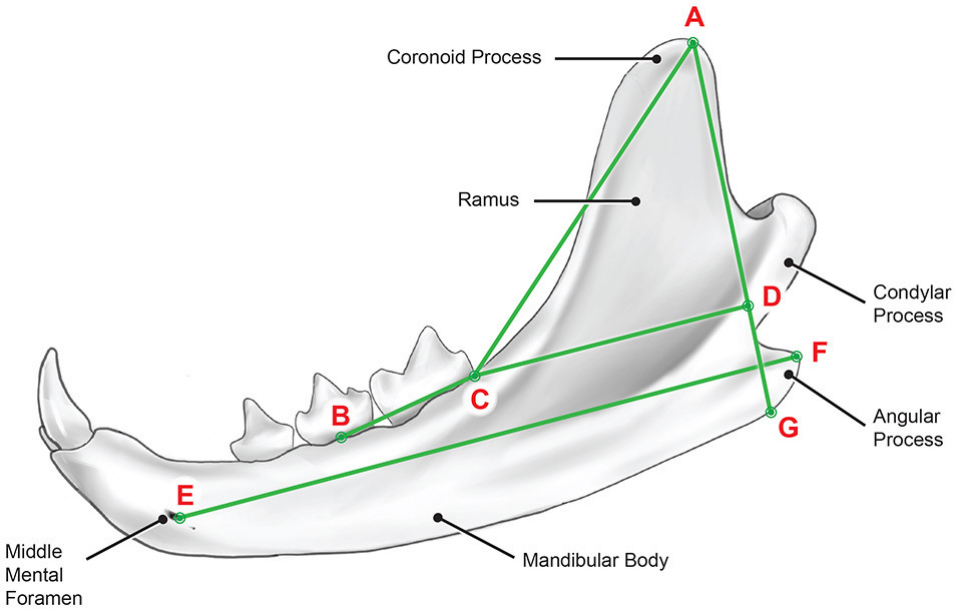
Diagram showing the reference points used for morphological measurement in this study.

The reference points used for morphological measurement are shown in Figure 2. In the coronal plane, the most dorsal point of the right and left coronoid process was identified and marked (A^r^) and (A^l^) respectively. The distance between (A^r^) and (A^l^) is the mandibular width at the coronoid process (WCP).

The MPR was re-orientated to obtain a sagittal oblique imaging plane which was parallel to the long axis of each mandible and parallel to the dorsoventral axis of the ramus. A MIP of each mandible was viewed separately with a slab thickness of 3mm. All subsequent landmarks were labeled with ^r^ and ^l^ to denote right and left respectively.

A line was drawn from (A) which intersected tangentially with the most rostral surface of the mandible between the condylar and angular processes and then with the ventral aspect of the mandible (G). The distance between (A) and (G) is the height of the ramus (RH). Point (G) was also marked.

A line was drawn from (A) to the most distal aspect of the first molar at the cemento-enamel junction (C). The distance between (A) and (C) is the ramus length (RL).

A line was drawn from (C) to intersect perpendicular with line (A-G) at (D). The distance between (C) and (D) is the ramus width (RW).

A line was drawn from the most caudal and lateral rim of the middle mental foramen (E) to the most caudal aspect of the angular process (F). The distance between (E) and (F) is the mandibular body length (MBL).

The furcation of the mandibular fourth premolar was marked (B) and a line drawn from this point to (C). The angle of intersection of lines (B-C) and (C-A) was measured and called the lateral ramus inclination angle (LRIA).

**Figure 3:**
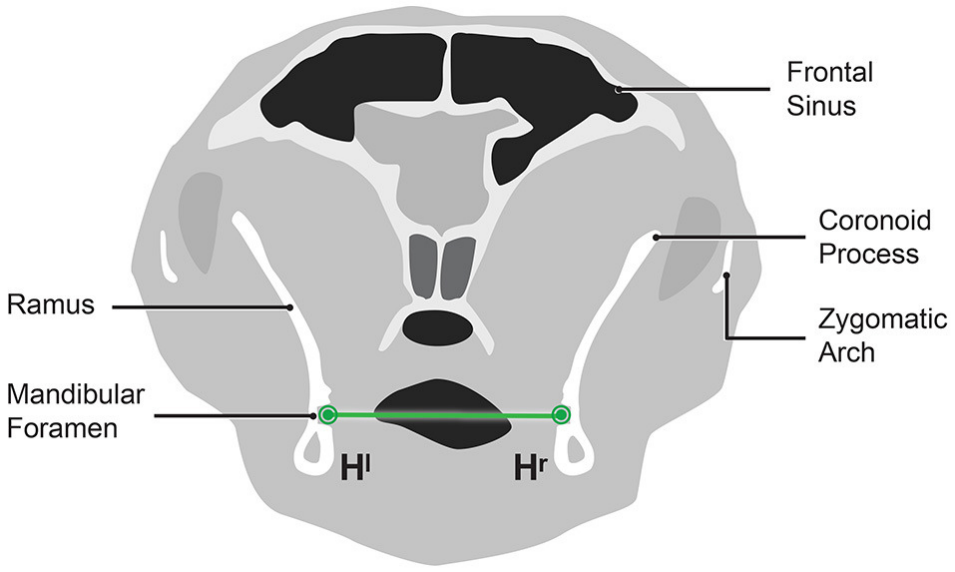
Diagramatic representation of an axial imaging plane CT image showing the width of the mandibles at the mandibular foramen (WMF).

The imaging plane was re-orientated to the axial plane image (Figure 3). The most rostral and medial rim of each mandibular foramen was marked (H^r^) and (H^l^). The distance between (H^r^) and (H^l^) is the width of the mandibles at the mandibular foramen (WMF).

**Figure 4:**
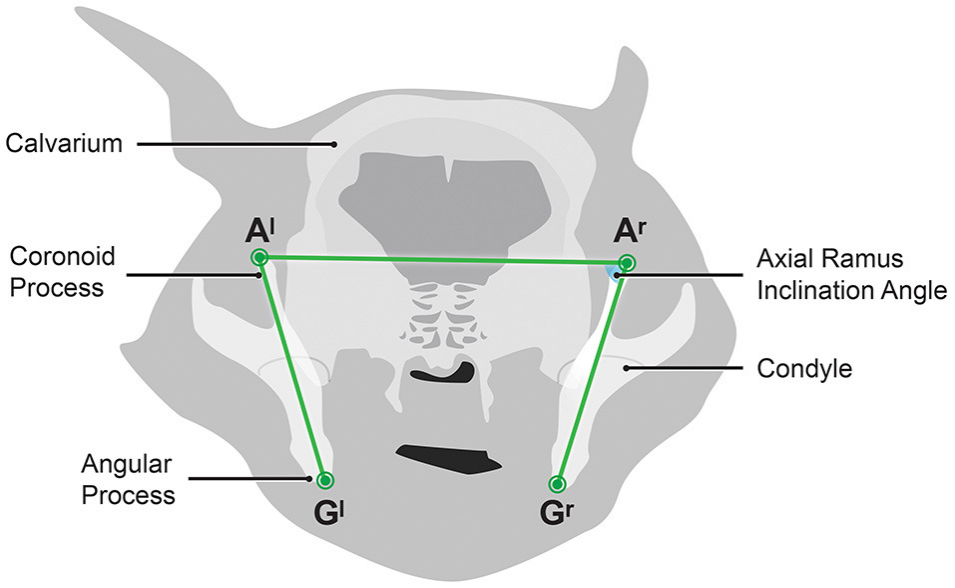
Diagramatic representation of an oblique axial imaging plane CT image showing the left and right axial ramus inclination angles.

The MPR was re-orientated to obtain an oblique axial imaging plane in which (A^r^), (A^l^), (G^r^) and (G^l^) could be viewed in the same plane (Figure 4). The angle of intersection of (G^r^-A^r^) and (A^l^-A^r^) was measured and called the right axial ramus inclination angle (ARIA^r^). The angle of intersection of (G^l^-A^l^) and (A^r^-A^l^) was measured and called the left axial ramus inclination angle (ARIA^l^).

Analysis of the data was performed. Using the measurements obtained the ratios between paired right and left measurements, and the ratio of jaw widths at the coronoid process and mandibular foramen (WCP:WMF), were calculated.

To perform the analysis, the right: left measurement ratios were modeled using a linear model to estimate the mean ratio and 95% confidence interval. The mean value was also compared to 1, perfect symmetry, using a *t*-test. Finally, the standard deviation was estimated to allow calculation of the coefficient of variation (CV) where the CV shows the amount of variation in the data proportional to the mean.

The degree of consistency in the morphometric measurements between cats was assessed using a second analysis. The right and left measurements for each cat were modeled using a linear model to estimate the mean value, 95% confidence interval and standard deviation which was used to calculate the CV.

Pearson's correlation coefficients were calculated for all possible pairs of measurements and tested for significance against 0. A *p*-value of less than 0.05 implies that the estimated correlation is significantly different from 0 (no correlation) and correlation is therefore present in the data. It is generally agreed that a correlation coefficient of greater than 0.7 indicates a strong positive correlation. The correlation between pairs of measurements of the right and left ramus was estimated in order to understand whether the change in osteometric measurement is proportional. For example, if the width of the mandible increases does the length and height increase proportionately.

All analyses were performed using R version 3.3.3 (16) and the *multcomp* library (9).

## Results

This retrospective study included 28 cats of which 14 were neutered male and 14 neutered females. The mean age was 104.3 months (range 8–222 months). The breed distribution was 26 Domestic Shorthaired, one Domestic Long Haired and one Burmese.

**Figure 5:**
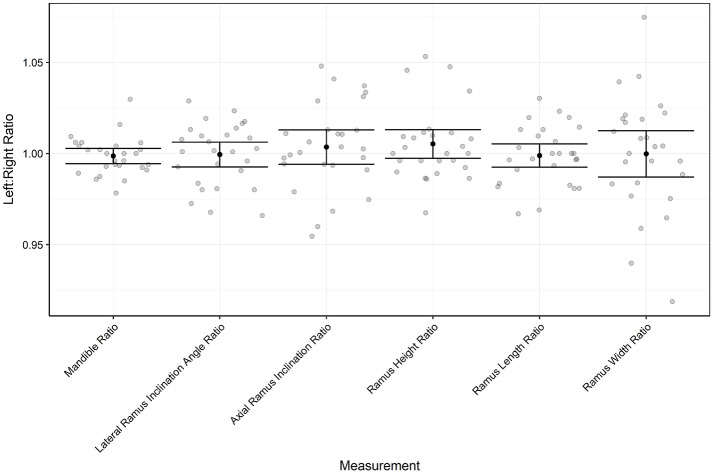
Shows the estimated mean ratios with 95% confidence intervals for all measurements.

The results of the symmetry analysis are shown in Table 1 and Figure 5 (Data Sheet 1). None of the right: left ratios were detected as being significantly different from 1 although this result alone doesn't imply symmetry. Alongside the lack of significant differences, the %CV values were all very small ranging from 1.038 to 3.637%. This shows that when cats deviate from the mean ratio they do so by only a small amount. The 95% confidence interval is the range that the mean will be within 95% of the time given the level of noise in the data. The 95% confidence interval is within 1% of the actual mean for all ratios apart from the ramus width ratio (1.4%).

**Table 1:** Symmetry analysis showing ratios of right and left measurements (SD, standard deviation; CI, confidence interval; %CV, %coefficient of variation).

**Mandible measure**	**Mean ratio**	**95% CI lower**	**95% CI upper**	***SD***	**% CV**	***p*-value**
Mandible Ratio (MBL^r^:MBL^l^)	1.00	0.99	1.00	0.01	1.05	0.495
Lateral Ramus Inclination Angle Ratio (LRIA^r^:LRIA^l^)	1.00	0.99	1.01	0.02	1.72	0.869
Axial Ramus Inclination Angle Ratio (ARIA^r^:ARIA^l^)	1.00	0.99	1.01	0.02	2.37	0.451
Ramus Height Ratio (A^r^-G^r^:A^l^-A^r^)	1.01	1.00	1.01	0.02	1.99	0.185
Ramus Length Ratio (A^r^-C^r^:A^l^-C^l^)	1.00	0.99	1.01	0.02	1.62	0.722
Ramus Width Ratio (C^r^-D^r^:C^l^-D^l^)	1.00	0.99	1.01	0.03	3.22	0.972

Measurements were then analyzed to determine how consistent individual measurements were between cats (Table 2). The mean for each measurement was calculated and from this the %CV which is an indicator of consistency or variation.

**Table 2:** Comparing osteometric measurements between cats (SD, standard deviation; CI, confidence interval; %CV, % coefficient of variation).

**Mandible measure**	**Region**	**Mean**	**95% CI lower**	**95% CI upper**	***SD***	**%CV**
Jaw width (cm)	CP	5.13	5.00	5.26	0.32	6.31
Jaw width (cm)	MF	3.03	2.94	3.13	0.24	8.02
Mandible length (cm)	R	5.05	4.91	5.18	0.33	6.61
Mandible length (cm)	L	5.05	4.91	5.19	0.34	6.65
LRIA (degrees)	R	145.59	144.35	146.84	3.16	2.17
LRIA (degrees)	L	145.70	144.28	147.13	3.61	2.48
ARIA (degrees)	R	76.68	75.43	77.93	3.16	4.12
ARIA (degrees)	L	76.44	75.11	77.77	3.37	4.41
Ramus height (cm)	R	2.76	2.67	2.85	0.22	8.01
Ramus height (cm)	L	2.75	2.66	2.84	0.22	8.04
Ramus length (cm)	R	3.21	3.12	3.29	0.22	6.94
Ramus length (cm)	L	3.21	3.12	3.30	0.22	6.76
Ramus width (cm)	R	2.46	2.40	2.52	0.16	6.32
Ramus width (cm)	L	2.46	2.40	2.52	0.16	6.29
Height:width	R	1.12	1.10	1.15	0.06	5.30
Height:width	L	1.12	1.09	1.15	0.07	6.23

The most consistent measurement was the lateral ramus inclination angle (LRIA) with a %CV of 2.196–2.463. The least consistent measurement was ramus height with a %CV of 8.089–8.259.

The correlation between pairs of measurements of the right and left ramus was analyzed and the results are shown in Table 3.

**Table 3:** Correlation between pairs of measurements of the right and left ramus.

**Contrast**	**Correlation**	***p*-value**	**Interpretation**
Height Ramus R - Width Ramus R	0.76	<0.001	Strong
Height Ramus L - Width Ramus L	0.67	<0.002	Moderate
Height Ramus R - Length Ramus R	0.83	<0.001	Strong
Height Ramus L - Length Ramus L	0.82	<0.001	Strong
Length Ramus R - Width Ramus R	0.85	<0.001	Strong
Length Ramus L - Width Ramus L	0.83	<0.001	Strong

## Discussion

This study shows that there is a high degree of mandibular symmetry in a sample of normal cats, with a small degree of variation in morphometric measurements between cats and that there is strong correlation between the height, width and length of the ramus in cats.

Three-dimensional (3D) analysis is essential for making an accurate assessment of craniofacial morphology allowing the detection of asymmetry (10). It has been shown that measurements obtained from CT 3D reconstruction provide an accurate representation of the craniofacial structures that have been scanned (13). This assessment cannot be performed accurately using conventional radiographs because of the superimposition of craniofacial bony structures and positioning errors during the acquisition of radiographs.

In this study, landmarks were chosen which were easily and consistently identifiable and included points which allowed morphometric analysis of the caudal part of the mandible. A study by Naji et al. (11) identified anatomic structures in three dimensions and examined their reliability to be used as landmarks in a three-dimensional co-ordinate cephalometric analysis using cone-beam computerized tomography (CBCT). The study demonstrated that a wide range of landmarks including teeth, foramina and the lateral aspect of the condyles of the mandible were reliable. For the data in this study to be useful in 3D cephalometric analysis further studies are required to demonstrate that there is intra and inter-observer reliability in identification of the chosen landmarks in cats.

Facial symmetry is defined as the correspondence in size, form and arrangement of the facial features on opposite sides of the median sagittal plane (15). There are many studies looking at facial symmetry in people but there are no equivalent studies in cats. Perfect correspondence in size, form and arrangement of the facial features on opposite sides of the face is unlikely and therefore this and other similar studies are measuring degrees of asymmetry. The coefficient of variation (CV) is defined as the ratio of the standard deviation to the mean and is often expressed as a percentage. The ratio of the right and left mandibular length (MBL^r^:MBL^l^) had the lowest %CV of 1.05 which represents 0.5 mm difference and the highest %CV was 3.22% for the ratio of right and left ramus width (C^r^-D^r^:C^l^-D^l^) which represents 0.9 mm. The %CV of the ratios associated with mandibular shape—Lateral Ramus Inclination Angle Ratio (LRIA^r^:LRIA^l^) and Axial Ramus Inclination Angle Ratio (ARIA^r^:ARIA^l^)—represented a difference of 2.5 and 1.8 degrees difference respectively. This demonstrates a very low level of asymmetry in both size and shape (Table 1).

The mandibles are commonly fractured in cats due to their prominent position and the incidence of serious trauma representing 14.5% of fractures in total (20). Injury can result from a road traffic accident (RTA), a bite from a dog or cat, or a fall from a height (high rise syndrome) (4).

Fractures of the caudal part of the mandible are often associated with malocclusion and stabilization is difficult (21). Techniques that have been advocated for the repair of caudal mandibular fractures include maxillo-mandibular fixation (MMF), interfragmentary wire, external fixators, bignathic encircling retention device (BEARD) and internal rigid fixation (IRF) using miniplates and screws (1, 2, 6, 12, 21). MMF and BEARD are techniques associated with a high degree of patient morbidity during the period of inter-arcade fixation. Problems encountered with these techniques include: difficulty with thermoregulation, long periods of hospitalization and assisted feeding, and an increased risk of aspiration of food or regurgitated stomach contents (2, 8, 12, 17, 21). Interfragmentary wiring is not suited to the thin, fragile bone of the feline ramus. Using this technique, it is difficult to position the fragments accurately and the very small cross-sectional surface area of the fracture surfaces results in insufficient stability of the repair during and after application of the wires (3). Similarly, much of the bone of the ramus is unsuitable for the use of external fixators due to the poor pin bone interface, leading to premature pin loosening and pull-out (1).

Ramus fractures in dogs and humans are frequently stabilized using internal rigid fixation (IRF) with plates and screws, providing accurate fracture reduction and good construct stability (1, 3). In cats this is complicated by their small size, the need for greater contouring of implants, difficulty in positioning small fragile fragments of bone during the application of implants, and the very small cross-sectional surface area of bone at the fracture sites making accurate anatomical reduction challenging. The advantages of IRF in the treatment of mandibular fractures are the accurate restoration of normal anatomy and occlusion and rapid return to normal function.

In humans, techniques have been developed to improve the accuracy and efficiency of complex craniofacial fracture repair including using a 3D model of the mirror image of the contralateral intact structure as a template for preoperative plate contouring (7). Custom implants for the reconstruction of craniofacial defects can be manufactured providing precise adaptation to the region of implantation, reduced surgical times and providing more accurate fracture reduction (14).

In our study no rami had as much as 5% asymmetry in height which corresponds to a difference in height of 1.2 mm between sides. This is unlikely to be of clinical significance when using the contralateral mandible as a template for plate contouring. Given this is the variable with the greatest coefficient it illustrates the level of symmetry that is present. Therefore, this study suggests that, in cats with a complex unilateral caudal mandibular fracture, a 3D model based on the mirror image of the intact contralateral mandible will be a suitable template for pre-contouring implants or for the manufacture of custom implants. It has also demonstrated that quantitative 3D measurement data from the contralateral intact mandible could be used as a template against which to evaluate the accuracy of mandibular fracture fixation techniques. This would be useful for the assessment of individual cases post-operatively and as a tool to allow the comparison of different techniques.

Comparison of specific measurements between cats demonstrates the level of variation for each parameter. This was also calculated as a %CV (Table 2). The most consistent measurement was the LRIA (%CV^l^ 2.17% and %CV^r^ 2.48%) and the least was the RH (%CV^l^ 8.01% and %CV^r^ 8.04%). The standard deviation therefore represents 3.5 degrees and 2.3 mm respectively. This represents a high degree of consistency between individuals. In fact, the %CV of the LRIA between cats of 2.17 and 2.48% for left and right mandibles respectively is very similar to the %CV of the ratio of the LRIA between the left and right sides of individual cats which was 1.72%.

This study also shows that there is an overall strong correlation between the height, width and length of the ramus in cats (Table 3). In other words, as the height of the ramus increases there is a proportional increase in its length and width.

The use of locking plates and screws in fracture fixation does not require the close contact between the bone and implant required when using neutralization plates and though the fixation should respect the bone axes and length it doesn't require precise anatomic reduction (5). The small variation in shape and size of mandibles between cats and strong correlation between mandibular dimensions in individual cats opens the possibility of developing a small range of standard pre-contoured locking plates for the fixation of caudal mandibular fractures.

The main limitation of this study is that the study population is of entirely domestic mixed breed cats. Whilst it seems likely that a similar low level of asymmetry will be present in other breeds it is possible that there will be a lower level of consistency in the dimensions of mandibles between cats with differing skull shapes such as brachycephalic cats, for example British Shorthair or Persian, and dolichocephalic cats, for example Abyssinian or Siamese. This may limit the use of standard pre-contoured plates to mesaticephalic cats or possibly domestic mixed breed cats. In order to evaluate this morphological analysis and inter-breed comparison of mandibular dimensions between cats with different skull shapes is required.

## Conclusion

This study has demonstrated both a low level of asymmetry between contralateral mandibles in cats and a high level of consistency in the dimensions of mandibles between cats.

This data provides clinically relevant evidence that use of a 3D model of the mirror image of the contralateral mandible is valid for planning fracture fixation and pre-contouring implants. It also provides clinically relevant data that can be used to assess the accuracy of fracture fixation. Demonstration of the consistency of the dimensions of mandibles in cats may facilitate the development of standardized pre-contoured locking plates for caudal mandibular fracture repair.

## Author contributions

PS and DB contributed to the design of this study, PS drafted the article, PS, RH, and DB analyzed and interpreted the data, DB revised the work critically for important intellectual content, RH carried out the statistical analysis.

### Conflict of interest statement

The authors declare that the research was conducted in the absence of any commercial or financial relationships that could be construed as a potential conflict of interest.
